# Global trends and future projections of migraine burden in children aged 5 to 14 from 1990 to 2050

**DOI:** 10.3389/fneur.2025.1641599

**Published:** 2025-09-05

**Authors:** Shuzhen Liao, Guifeng Qian, Huaqing Huang, Haishou Fu

**Affiliations:** ^1^Department of Laboratory Medicine, Fuzhou University Affiliated Provincial Hospital, School of Medicine, Fuzhou University, Shengli Clinical Medical College of Fujian Medical University, Fuzhou, Fujian Province, China; ^2^Department of Rehabilitation Medicine, Foshan Hospital of Traditional Chinese Medicine, Foshan, China; ^3^Department of Pain Medicine, Clinical Oncology School of Fujian Medical University, Fujian Cancer Hospital, Fuzhou, Fujian Province, China

**Keywords:** migraine, global burden of disease, incidence, Bayesian age-period-cohort model, headache

## Abstract

Migraine is a leading cause of disability, yet its burden in children aged 5 to 14 remains underexplored. Using Global Burden of Disease (GBD) 2021 data, we analyzed global, regional, and national migraine burdens from 1990 to 2021. Age-standardized incidence (ASIR), age-standardized prevalence (ASPR), and disability-adjusted life years (DALY) were assessed, with estimated annual percent change (EAPC) used for trend evaluation. A Bayesian Age-Period-Cohort (BAPC) model projected incidence to 2050. Globally, migraine cases rose by 39.5% (21.95 to 26.87 million), though ASIR, ASPR, and DALY rates remained stable. Low-middle Sociodemographic Index (SDI) regions had the highest prevalence (31.19 million) and DALY (1.11 million), while high SDI regions recorded the highest female incidence rates. Western Sub-Saharan Africa showed the largest increases across all metrics (EAPCs > 3.0). Brazil reported high ASIR and DALY, while Thailand had the greatest DALY reduction, likely due to effective public health measures. Projections suggest declining ASIR, ASPR, and DALY rates by 2050. These findings indicated absolute cases of migraine have increased due to population growth, the age-standardized burden has remained stable over time. Strengthened surveillance, targeted screening in low-middle SDI regions, and school-based awareness programs in high-burden countries are essential to mitigating migraine-related disability in this vulnerable population.

## Introduction

Migraine is a common and debilitating neurological disorder, characterized by recurrent episodes of moderate-to-severe headache, typically accompanied by multisystem dysregulation including nausea, photophobia, and phonophobia (1, 2). Some patients experience aura phenomena—transient focal neurological symptoms preceding headache onset—that may involve visual, sensory, or language disturbances (2). As the second most prevalent neurological disease globally, migraine affects approximately 14–15% of individuals, with a disproportionately high burden observed in females attributable to hormonal fluctuations and sex-specific pain processing mechanisms (3, 4). Notably, it ranks as the fifth leading cause of disability for women and the 20th for men, according to the Global Burden of Disease (GBD) study (1). Although migraine predominantly affects adults, its prevalence among children and adolescents is increasingly recognized (5, 6). Previous meta-analyses have shown that the global prevalence of migraine in children and adolescents reaches approximately 11%, with higher rates observed in adolescents and in girls (7). Migraine demonstrates significant cognitive associations, particularly migraine without aura showing consistent links to impaired memory and executive function through standardized cognitive assessments (8). Early-onset migraine emerges as an independent risk factor for accelerated cognitive decline, highlighting critical intervention windows during pediatric/adolescent stages (9). In younger populations, migraine disrupts daily activities, hinder educational performance, and reduce quality of life, underscoring the need for targeted public health interventions to mitigate their long-term effects (2, 10).

The GBD study offers a robust framework to quantify health loss across populations and over time, utilizing standardized methodologies and an expansive dataset encompassing over 607 billion systematically curated data points from 204 territories, provides unparalleled capacity for cross-temporal and cross-spatial health loss quantification (11). This analytical framework employs disability-adjusted life years (DALY)—a composite metric integrating years of life lost (YLLs) and years lived with disability (YLDs)—to enable comparative burden assessment across 369 diseases and injuries (1, 12). Particularly relevant to migraine research, the GBD’s granular stratification by age (5-year intervals), sex, and geographical location enables detailed analysis of epidemiological patterns across specific demographic groups and regions, which is valuable for informing targeted public health approaches, while acknowledging the limitations inherent in these model-based estimates derived from diverse sources and imputation methods. Its ability to generate age-, sex-, and location-specific data enables nuanced analyses of epidemiological patterns, making it an invaluable tool for tailoring health policies and interventions to address the global and regional burden of migraine in specific demographics (11). The GBD framework’s focus on evidence-based decision-making aligns with evidence-informed policy-making, essential for effective health policies. By offering robust data on health trends and risks, it helps policymakers make science-based decisions, crucial in resource-limited settings to maximize intervention impact (13).

Despite growing recognition of pediatric migraine, its global disease burden remains poorly characterized (14). Building on GBD 2021 data, this study aimed to evaluate the incidence, prevalence, and DALY associated with pediatric migraine from 1990 to 2021, with an analysis of projected trends through 2050. By examining temporal and regional disparities in pediatric migraine burden, this study provides actionable insights to inform the design of age- and context-specific health policies, optimize resource allocation, and guide early intervention strategies aimed at improving long-term outcomes in affected children.

## Methods

### Data source

Migraine data for children aged 5–14 years were obtained from the GBD 2021, which provides comprehensive estimates for 369 diseases, injuries, and impairments, as well as 88 risk factors across 204 countries and territories grouped into 21 regions (15). Data were retrieved from the Global Health Data Exchange GBD Results Tool (https://vizhub.healthdata.org/gbd-results/).

Migraine is classified as a disabling primary headache disorder, commonly characterized by recurrent, moderate to severe unilateral pulsatile headaches. According to the International Classification of Diseases (ICD), migraine is coded within the range of 346–346.93 in ICD-9 and under the codes G43–G43.919 in ICD-10 (16–18). Specifically, migraine was identified as a distinct entity at level 4 within the headache disorders group (level 3), which is categorized as the third level under the broader categories of neurological disorders (level 2) and non-communicable diseases (level 1).

The GBD 2021 classification divides the world into 21 geographic regions based on epidemiological similarities and geographic proximity, allowing for nuanced insights into variations in the disease burden and facilitating the development of targeted public health policies and interventions (19, 20). The 21 regions in the GBD 2021 classification encompass diverse areas, including Andean Latin America, Australasia, the Caribbean, Central Asia, Central and Eastern Europe, and Latin America. It also covers regions from Sub-Saharan Africa, such as Central, Eastern, and Southern Sub-Saharan Africa, alongside high-income regions like Asia Pacific and North America. Additionally, regions from the Middle East, Oceania, South Asia, Southeast Asia, and various parts of Latin America, such as Southern Latin America and Tropical Latin America, were included. Furthermore, Western Europe and Western Sub-Saharan Africa are also represented. This classification system has been consistently applied in previous GBD iterations and remains effective in comparing health metrics across distinct areas (11, 12).

The GBD 2021 provided data on incidence rates, prevalence rates, DALY rates, incident cases, and prevalent cases of migraine. The rates were age-standardized and expressed per 100,000 individuals. To calculate the 95% uncertainty intervals (UI), the GBD generated 1,000 estimates for each value using their analytical modeling approach (20). The values at the 25th and 975th rank positions among these 1,000 ordered estimates were then utilized to define the lower and upper bounds of the 95% UI, respectively. The methodological approach employed in GBD 2021 has been documented in prior related literature (21, 22).

The sociodemographic index (SDI) (23) is a joint assessment of the local socioeconomic environment by combining information on lagged distributions of per capita income, the average educational attainment among individuals aged 15 years and older, and the total fertility rate among individuals younger than 25 years (24). In the GBD Study 2021, each geographic location has its corresponding SDI value, based on which the study team separated 204 countries and territories into five groups, including low (< 0.46), low-middle (0.46 ~ 0.60), middle (0.61 ~ 0.69), high-middle (0.70 ~ 0.81), and high (> 0.81) (25).

The estimated annual percentage change (EAPC) was calculated using joinpoint regression:

EAPC = [exp(*β*) - 1] × 100%, where *β* represents the slope coefficient from log-linear model:

In(ASR)=a+βX+e . Linearity assumption was verified through cumulative sum (CUSUM) tests, with sensitivity analysis comparing linear vs. spline regression models. This approach is preferred for its capacity to: (1) quantify temporal trends in unevenly spaced observations; (2) enable cross-country comparability; (3) accommodate sparse data through empirical Bayes smoothing (26, 27).

Age-standardized rates (ASR) were computed per 100,000 population using the direct method: ASR=(Σ(wi×ai))/Σwi×100,000 , where ai = age-specific rate, wi = GBD global standard population weights. Uncertainty intervals (UI) were generated via 1,000 Monte Carlo simulations, with convergence confirmed by Gelman-Rubin statistic (28).

We employed a Bayesian age-period-cohort (BAPC) model to project the age-standardized incidence, prevalence, and DALYs of migraine through 2050. This model incorporates age, period, and cohort effects while assuming linear trends across time. The BAPC model was chosen due to its interpretability, transparency, and successful application in previous global disease burden studies. Although it assumes additive linearity across components, it can accommodate moderate deviations from linearity through Bayesian smoothing and random walk priors, enabling a balance between model flexibility and computational stability. For projections to 2050, BAPC modeling was implemented: log(μ_{a,p})=α_a+β_p+γ_c+ε , where:α_a = age-specific random walk prior (RW1), β_p = period effects with shrinkage priors (N(0,τ^2^)), γ_c = cohort effects via intrinsic conditional autoregressive (ICAR) structure (29). We employed the BAPC model to forecast the incidence of migraine among individuals aged 5–14 years old for 2050.

### Statistical analysis

First, we incorporated migraine data for two age groups: 5–9 years and 10–14 years. We then described the numbers and trends of incident cases, prevalent cases, and DALY for migraine, along with their corresponding ASR and associated 95% UI. These metrics were stratified by sex, age, year, SDI subregion, 21 regions, and 204 countries and territories. The BAPC model was used to project the number and rate of migraine-related disease burden from 2022 to 2050. Figures were created using R software (version 4.2.3) and JD_GBDR (V2.25, Jingding Medical Technology Co., Ltd.). Statistical significance was defined as *p* < 0.05.

## Results

### Global level

**Figure 1:**
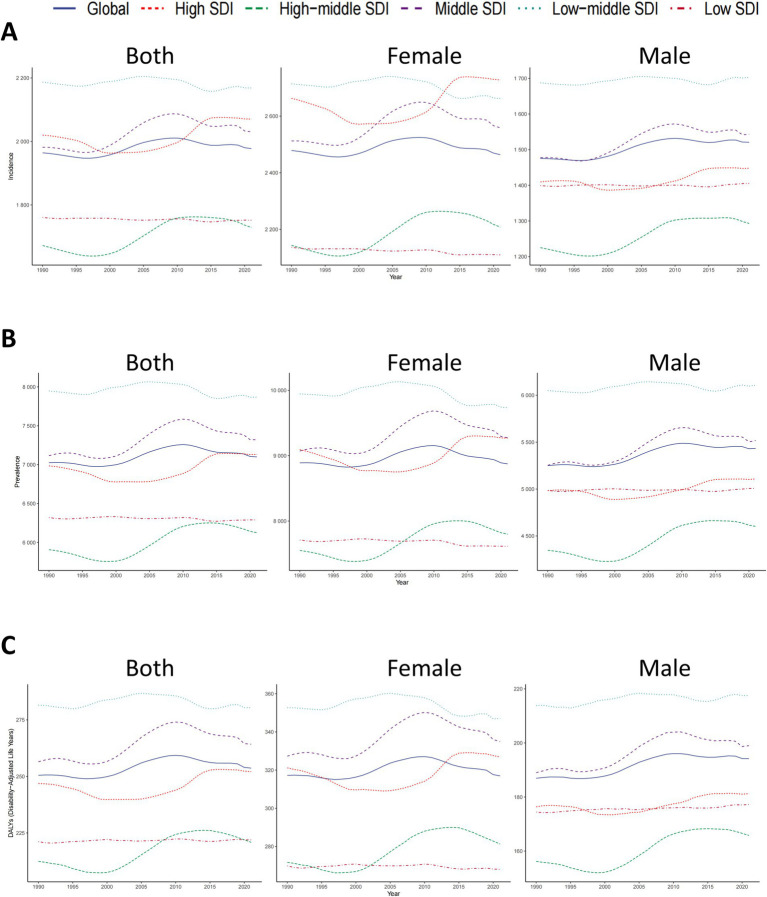
Trends in migraine prevalence, incidence and disability-adjusted life-years from 1990 to 2021.

Globally, the incidence, prevalence, and DALY of migraine among children increased significantly (Figure 1; Table 1; Supplementary Tables S1,S2). From 1990 to 2021, individuals aged 5–14 experienced a substantial increase in migraine burden. By 2021, the estimated incidence climbed to 26.87 million cases (95% UI: 19.04–35.96 million), marking a 39.52% rise from the 21.95 million cases (95% UI: 15.49–29.47 million) observed in 1990. The global age-standardized incidence rate (ASIR) increased slightly from 1,964.27 per 100,000 in 1990 to 1,977.40 per 100,000 in 2021. Concurrently, prevalence cases surged by 24.50%, increasing from 78.26 million in 1990 to 97.43 million in 2021, while DALY showed a comparable growth of 24.73%, reaching 3.48 million in 2021 from 2.79 million in 1990.

**Table 1:** Incidence of migraine between 1990 and 2021 in 5 to 14 years at the global and regional level.

Characteristics	1990		2021		1990–2021
	Incidence cases (95%UI)	ASIR (per100,000) (95%UI)	Incidence cases (95%UI)	ASIR (per100,000) (95%UI)	EAPC of ASIR (95% CI)
Global	21954881.41 (15486594.86, 29472228.75)	1964.27 (1386.10, 2636.05)	26873086.22 (19043842.16, 35959617.35)	1977.40 (1399.69, 2648.34)	0.09 (−0.37, 0.55)
Gender					
Females	13501536.26 (9526069.75, 18134245.78)	2478.59 (1749.27, 3328.29)	16210855.94 (11460059.56, 21753645.94)	2463.66 (1739.61, 3308.74)	0.04 (0.00, 0.07)*
Males	8453345.15 (5930535.10, 11453311.95)	1475.43 (1035.55, 1998.28)	10662230.29 (7530927.12, 14393182.45)	1520.84 (1073.20, 2054.93)	0.15 (0.12, 0.18) *
Sociodemographic index					
High SDI	2522682.72 (1735939.50, 3436463.46)	2020.00 (1388.30, 2754.53)	2486488.14 (1711443.13, 3410861.63)	2070.49 (1421.62, 2844.91)	0.14 (−0.43, 0.71)
High-middle SDI	3041461.58 (2119366.54, 4099594.80)	1672.08 (1163.31, 2256.50)	2788368.45 (1945558.91, 3768773.45)	1729.17 (1205.84, 2338.21)	0.28 (−0.22, 0.79)
Middle SDI	7475395.93 (5341877.13, 9957291.47)	1981.69 (1415.53, 2640.51)	7959845.70 (5670469.78, 10578536.27)	2030.23 (1444.45, 2700.91)	0.18 (−0.29, 0.66)
Low-middle SDI	6491075.72 (4605176.92, 8692214.39)	2186.92 (1554.32, 2923.76)	8462456.48 (6058000.40, 11269846.65)	2168.42 (1549.56, 2891.88)	−0.02 (−0.44, 0.41)
Low SDI	2403982.83 (1662866.25, 3257151.06)	1760.78 (1222.75, 2378.73)	5155048.29 (3595953.07, 6987976.68)	1751.68 (1222.41, 2373.81)	−0.02 (−0.39, 0.36)
21 GBD regions					
Andean Latin America	176154.35 (125473.48, 237070.34)	1840.23 (1310.59, 2476.81)	225910.27 (156290.85, 314659.50)	1889.60 (1305.92, 2634.38)	0.12 (−0.52, 0.76)
Australasia	52958.59 (35201.68, 73669.76)	1724.93 (1144.70, 2402.71)	68169.25 (45321.89, 94822.17)	1723.92 (1144.00, 2401.60)	0.00 (−0.37, 0.37)
Caribbean	189069.78 (130785.12, 261665.95)	2593.11 (1791.80, 3592.67)	197717.75 (136846.92, 273413.71)	2582.80 (1784.40, 3578.20)	−0.00 (−0.57, 0.56)
Central Asia	271542.98 (179784.31, 388651.55)	1766.82 (1172.02, 2524.94)	308321.04 (204099.29, 441401.09)	1755.84 (1164.69, 2509.62)	−0.01 (−0.53, 0.52)
Central Europe	359803.81 (246879.56, 493713.95)	1741.57 (1190.37, 2396.68)	213935.61 (146765.38, 293073.72)	1737.30 (1186.76, 2387.79)	−0.01 (−0.53, 0.52)
Central Latin America	990018.78 (720212.41, 1319691.76)	2390.75 (1737.93, 3188.56)	1052045.99 (756075.59, 1405358.28)	2414.15 (1729.81, 3231.19)	0.06 (−0.53, 0.64)
Central Sub-Saharan Africa	243580.56 (160523.46, 343777.13)	1654.35 (1094.88, 2328.15)	618465.80 (408627.50, 871458.50)	1652.68 (1093.79, 2326.06)	−0.00 (−0.37, 0.37)
East Asia	2785312.14 (1937778.46, 3775698.92)	1291.88 (897.74, 1753.35)	2576080.11 (1803715.38, 3519727.96)	1381.21 (967.60, 1885.98)	0.32 (−0.21, 0.85)
Eastern Europe	565567.99 (395449.85, 764915.46)	1653.19 (1155.99, 2235.79)	420582.90 (294467.77, 568826.80)	1653.53 (1156.70, 2238.13)	0.01 (−0.51, 0.54)
Eastern Sub-Saharan Africa	584498.33 (397061.13, 809068.54)	1084.00 (738.26, 1497.65)	1256552.40 (853672.33, 1741741.12)	1096.60 (745.08, 1519.89)	0.06 (−0.31, 0.44)
High-income Asia Pacific	363486.68 (249950.72, 500528.13)	1421.67 (973.66, 1966.70)	226740.81 (156745.82, 313104.92)	1396.91 (963.14, 1933.89)	−0.05 (−0.61, 0.52)
High-income North America	880761.60 (606780.82, 1198321.45)	2195.41 (1511.84, 2988.49)	976659.57 (674915.64, 1339529.70)	2120.46 (1460.99, 2916.81)	0.01 (−0.66, 0.68)
North Africa and Middle East	2105794.99 (1476294.21, 2873327.69)	2367.75 (1663.24, 3226.47)	2989133.76 (2125485.38, 4034697.91)	2446.10 (1739.23, 3301.87)	0.13 (−0.15, 0.41)
Oceania	31190.76 (20868.12, 43774.41)	1870.30 (1253.36, 2622.15)	58494.98 (39129.74, 82102.58)	1869.37 (1252.86, 2620.70)	−0.00 (−0.39, 0.39)
South Asia	5821996.51 (4113906.75, 7872191.95)	2124.99 (1504.58, 2867.98)	7422999.12 (5268196.21, 9882178.56)	2107.81 (1491.79, 2812.90)	−0.05 (−0.49, 0.39)
Southeast Asia	2414844.85 (1660053.63, 3291409.99)	2143.47 (1472.96, 2922.43)	2477063.01 (1698208.99, 3377647.82)	2111.45 (1445.45, 2882.73)	−0.04 (−0.46, 0.38)
Southern Latin America	144672.17 (96166.95, 204808.77)	1474.24 (979.22, 2088.47)	153744.92 (101804.52, 216750.06)	1494.24 (987.93, 2109.11)	0.07 (−0.47, 0.62)
Southern Sub-Saharan Africa	217854.76 (150533.06, 297273.41)	1651.16 (1141.31, 2252.40)	265158.28 (183212.86, 362104.84)	1646.23 (1136.45, 2249.91)	−0.00 (−0.37, 0.36)
Tropical Latin America	1423371.83 (1076150.16, 1826224.78)	3895.96 (2943.47, 5002.24)	1354146.76 (1034706.91, 1737543.14)	4106.94 (3133.71, 5276.20)	0.42 (−0.12, 0.97)
Western Europe	1229248.55 (840463.78, 1682332.74)	2527.92 (1722.15, 3468.17)	1197645.46 (819236.55, 1639402.58)	2524.95 (1721.57, 3464.10)	0.03 (−0.53, 0.59)
Western Sub-Saharan Africa	1103151.40 (762615.50, 1495195.23)	2139.99 (1486.20, 2892.69)	2813518.44 (1959846.98, 3835278.98)	2097.77 (1463.96, 2856.12)	−0.06 (−0.41, 0.29)

EAPC, estimated annual percentage change. *Indicates significant trend.

**Figure 2:**
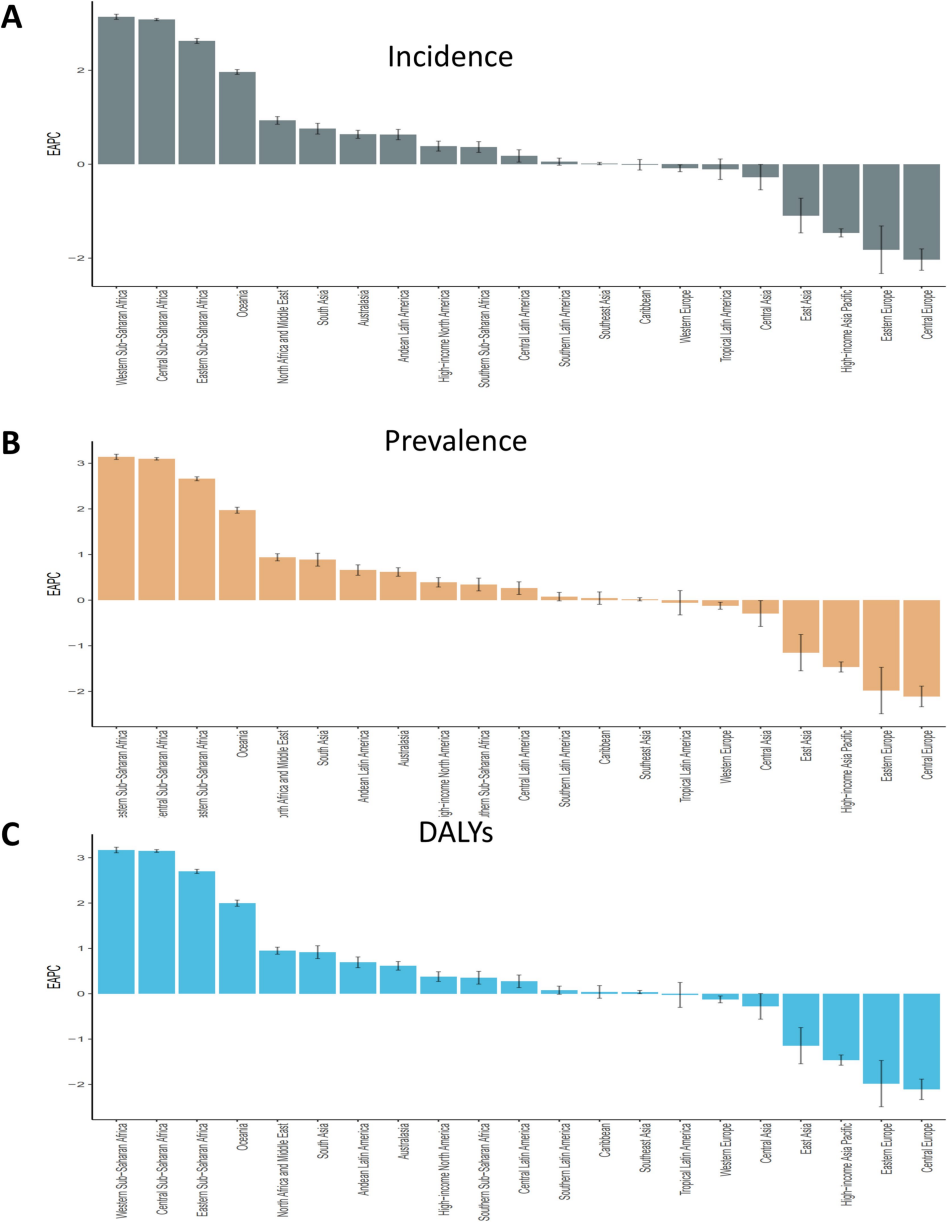
EAPC of migraine prevalence, incidence, and disability-adjusted life years cases across 21 GBD regions in both females and males. EAPC, estimated annual percentage change; GBD, global burden of disease.

Between 1990 and 2021, the global ASIR, ASPR, and age-standardized DALY rates of migraine among children aged 5–14 years remained relatively stable, with only minimal variations (Figure 1). The EAPC were 0.09 [95% Confidence Interval (CI): −0.37-0.55] for ASIR, 0.11 (95% CI: −0.36-0.58) for ASPR, and 0.12 (95% CI: −0.35-0.59) for age-standardized DALY rate (Figure 2; Table 1; Supplementary Tables S1,S2). While females consistently exhibited higher ASIR, ASPR, and age-standardized DALY rates, the overall global disease trends for both males and females followed similar patterns (Figure 1; Table 1; Supplementary Tables S1,S2).

### SDI regional level

In 2021, the Low-middle SDI region accounted for the highest migraine burden among children, with the highest prevalence (31,187,015.92 cases; 95% UI: 22,933,453.57-41,715,086.01), incidence (8,462,456.48 cases; 95% UI: 6,058,000.40-11,269,846.65), and DALY (1,112,359.59; 95% UI: 38,801.23-2,822,909.34) (Table 1; Supplementary Tables S1,S2). For females, High SDI regions exhibited the highest incidence rates, while Low-middle SDI regions had the highest prevalence and DALY rates. In contrast, the Low SDI regions had the lowest rates across all three metrics. For males, the Low-middle SDI regions displayed the highest prevalence, incidence, and DALY, whereas the High-middle SDI regions had the lowest rates (Figure 1).

### Regional level

South Asia was identified as the region with the highest migraine incidence, with an estimated 7,422,999.12 cases (95% UI: 5,268,196.21-9,882,178.56), while Oceania reported the lowest incidence, with 58,494.98 cases (95% UI: 39,129.74-82,102.58) (Table 1, Supplementary Tables S1,S2). Over the past three decades, Western Sub-Saharan Africa experienced the most dramatic increase in migraine burden, with statistically significant EAPCs of 3.14 (95% CI: 3.08–3.19) for incidence, 3.14 (95% CI: 3.08–3.20) for prevalence, and 3.17 (95% CI: 3.11–3.23) for DALY (Figure 2). In contrast, Central Europe saw the steepest decline in incidence (EAPC: -2.03; 95% CI: −2.26 to −1.80), prevalence (EAPC: -2.11; 95% CI: −2.34 to −1.88) and DALY (EAPC: -2.11; 95% CI: −2.33 to −1.88) (Figure 2; Supplementary Table S3). Tropical Latin America reported the highest ASIR at 4,106.94 per 100,000 population (95% UI: 3,133.71-5,276.20), the highest ASPR at 16,135.87 per 100,000 population (95% UI: 12,355.20-20,844.71) and the highest age-standardized DALY rate at 589.87 per 100,000 population (95% UI: 10.01–1,487.26), while Eastern Sub-Saharan Africa had the lowest rates (Table 1; Supplementary Tables S1,S2).

**Figure 3:**
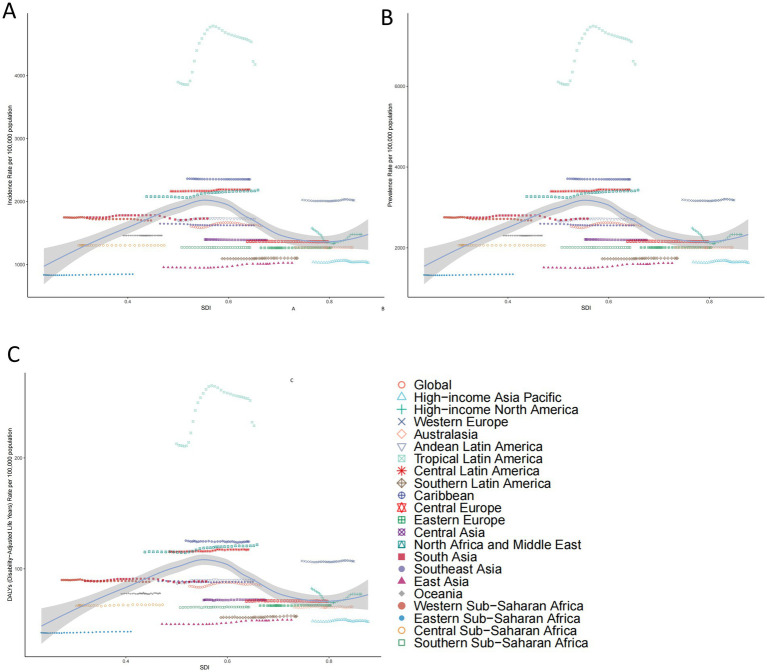
The associations between the sociodemographic index and migraine across 21 GBD regions. **(A)** Association between age-standardized migraine incidence rate and sociodemographic index. **(B)** Association between age-standardized migraine prevalence rate and sociodemographic index. **(C)** Association between age-standardized migraine DALY rate and sociodemographic index. GBD, global burden of disease; DALY, disability-adjusted life years.

In 2021, the ASIR, ASPR, and age-standardized DALY rate for migraine demonstrated a slight but statistically significant negative correlation with the SDI across 21 regions, demonstrating a rapid decline as the SDI increased (ASIR: *r* = − 0.069, *p* < 0.001; ASPR: *r* = − 0.127, *p* < 0.001; DALY: *r* = − 0.121, *p* < 0.001. Figure 3). In 2021, regions such as Western Sub-Saharan Africa, Southeast Asia, South Asia, Western Europe, Central Latin America, North Africa and the Middle East, Caribbean, and Tropical Latin America consistently show higher-than-global average rates for migraine incidence, prevalence, and DALY.

### Country-level analysis

**Figure 4:**
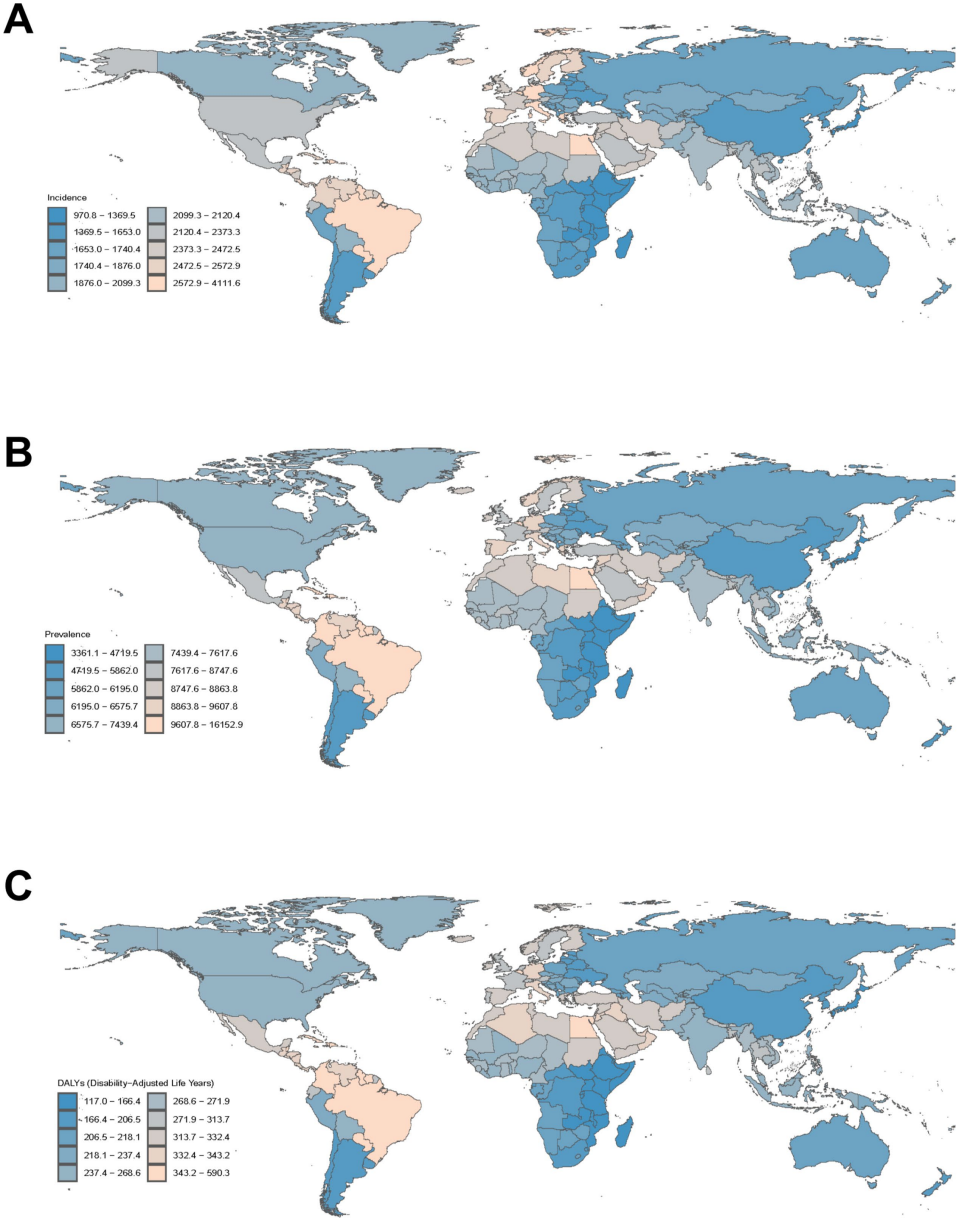
**(A)** The global disease burden of migraine ASIR for both sexes in 204 countries and territories. **(B)** The global disease burden of migraine ASPR for both sexes in 204 countries and territories. **(C)** The global disease burden of migraine ASDR for both sexes in 204 countries and territories. ASIR, age-standardized incidence rate; ASPR, age-standardized prevalence rate; ASDR, age-standardized disability-adjusted life years rate.

The global landscape of migraine incidence among individuals aged 5–14 years revealed significant disparities across nations. In 2021, Brazil emerged as the country with the highest ASIR of migraine, recording 4,111.61 cases per 100,000 population (95% UI: 3,131.84-5,274.07). In contrast, Ethiopia reported the lowest ASIR of 970.78 cases per 100,000 population (95% UI: 680.83–1,322.63) (Figure 4A; Supplementary Table S4). Qatar experienced a 318% increase in absolute case numbers (95% CI: 297–340%), followed by Angola (248%; 95% CI: 230–265%), while Albania showed a decline of 57.4% (95% CI: 54.2–60.1%) (Supplementary Figure S1A; Supplementary Table S4). Norway exhibited the most pronounced increase in migraine incidence (EAPC: 1.36; 95% CI: 1.07–1.64) (Supplementary Figure S2A; Supplementary Table S4). India, China, and Brazil accounted for the highest migraine prevalence among children aged 5–14 years in 2021(Supplementary Figure S1B; Supplementary Table S5). Brazil exhibited the highest ASPR of 16,152.9 per 100,000 population (95% UI: 12,355.2–20,844.7). Norway demonstrated the most significant increase in migraine prevalence (EAPC: 1.38; 95% CI: 1.09–1.68), whereas Thailand experienced the largest decrease (EAPC: -0.29; 95% CI: −0.37–-0.21) (Supplementary Figure S2B; Supplementary Table S5). For DALY, Brazil reported the highest age-standardized rate of 590.32 per 100,000 population (95% UI: 452.11–758.49) in 2021 (Figure 4; Supplementary Figure S1C; Supplementary Table S6). Norway exhibited the most marked increase in age-standardized DALY rates (EAPC: 1.39; 95% CI: 1.09–1.69) (Supplementary Figure S2C; Supplementary Table S6).

### Age and sex patterns

**Figure 5:**
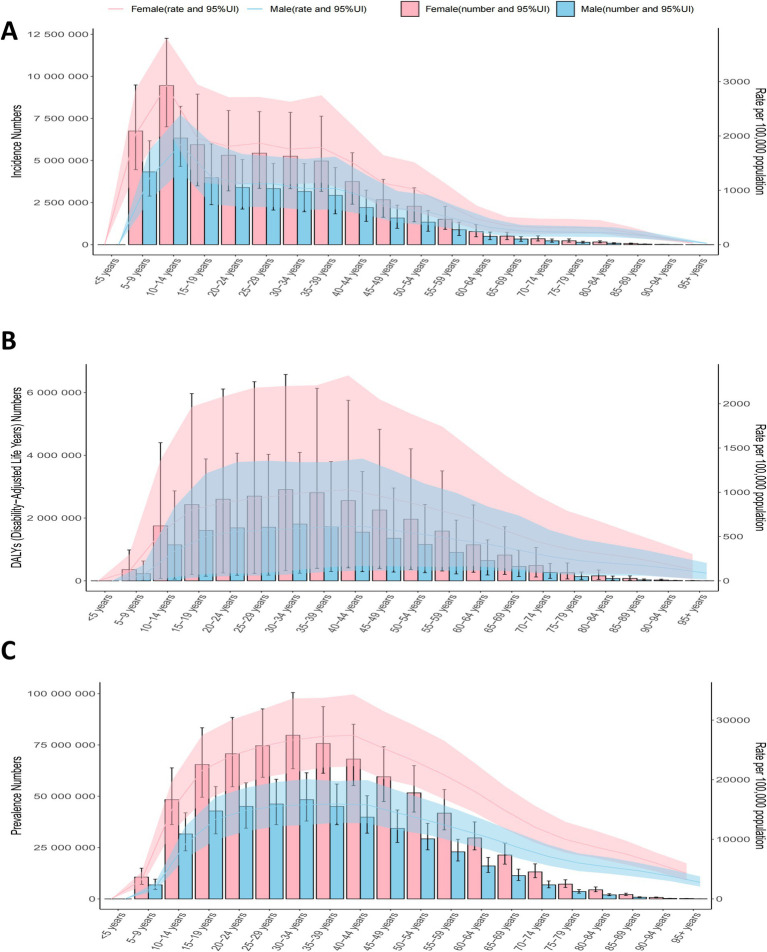
The number of cases and standardized rates of global migraine prevalence, incidence, and disability-adjusted life years (DALY) by age groups in 2021.

In 2021, the global distribution of migraine has revealed distinct patterns influenced by age and sex. Across all age ranges, females consistently exhibited higher incidence rates than males (Figure 5).

Additionally, the 10–14 age group exhibited the highest ASIR, with the 5–9 age group ranking second (Figure 5). While ASIR remained relatively stable across age groups, the absolute number of cases peaked in the 10–14 age group and subsequently declined with advancing age (Figure 5). Prevalence rates increased with age, peaking in the 30–34 age group (Figure 5). The DALY mirrored the prevalence trends. Across all ages, females consistently had higher ASPR, ASIR, and DALY rates than males, underscoring the significant gender disparity in the global migraine burden (Figure 5).

### Future burden of migraine

**Figure 6:**
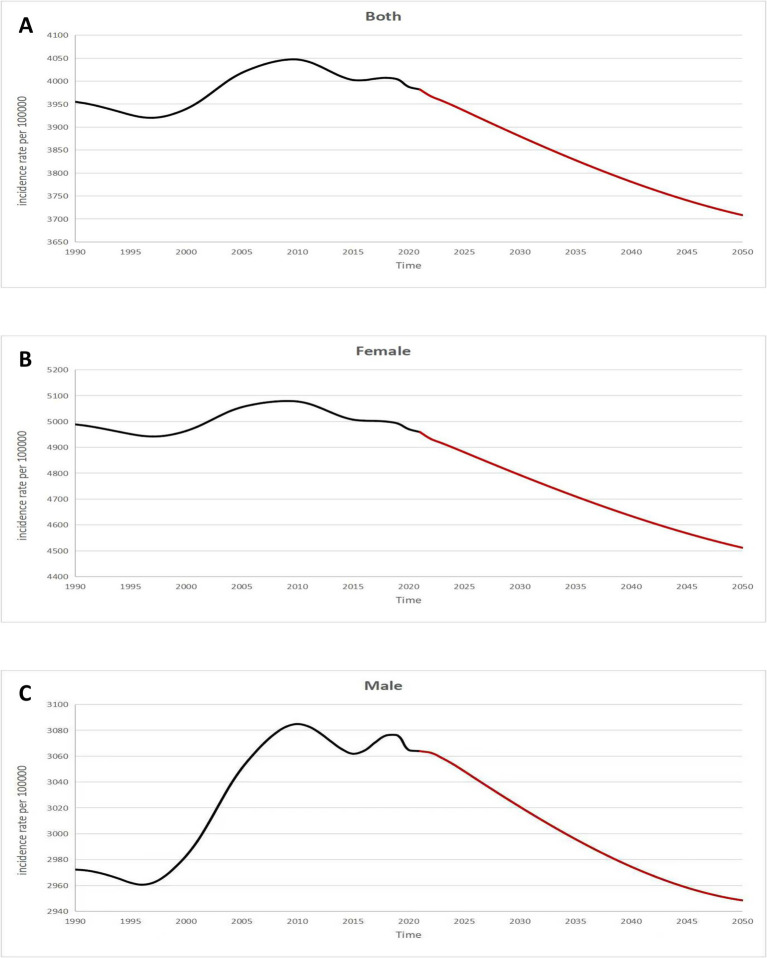
Future forecasts of GBD in migraine age-standardized incidence rate. GBD, global burden of disease.

Figure 6 depicts the projected global trends in migraine ASIR among individuals aged 5–14 years, indicating a continuous decline worldwide. By 2050, the global ASIR for migraine is expected to decrease to approximately 3,700 per 100,000 population (Figure 6). While women are projected to maintain higher ASIR levels compared to men (4,500 vs. 2,950 per 100,000), both sexes are anticipated to experience downward trends (Figure 6).

## Discussion

This study provides a comprehensive assessment of migraine burden among children aged 5–14 years using GBD 2021 data. Between 1990 and 2021, global migraine incidence increased by 39.5%, with cases rising from 21.95 million (95% UI: 15.49–29.47 million) to 26.87 million (95% UI: 19.04–35.96 million). Similar growth patterns emerged in prevalence (78.26 to 97.43 million) and DALY (2.79 to 3.48 million), though age-standardized rates remained stable (ASIR EAPC = 0.09), suggesting population growth rather than epidemiological shifts drove case escalation.

The observed developmental trajectory of migraine aligns with a growing body of literature documenting age-related increases in migraine prevalence and symptom changes during childhood and adolescence. A comprehensive population-based review highlighted a substantial rise in migraine prevalence between early childhood and adolescence, with prevalence rates increasing from approximately 3% at age 3 to over 20% by late adolescence (30). Migraine presentations show age- and sex-specific variations, with older children, particularly girls, often experiencing increased prevalence and more complex symptoms, including longer headache duration and heightened photophobia (7, 31). These findings are consistent with our GBD-based analysis, which shows rising incidence and burden metrics peaking in the 10–14 age group. We retain the Hungarian cohort study (32) as a longitudinal example that supports these trends at the individual level, but we now contextualize it within this broader international evidence base to provide a more comprehensive understanding of pediatric migraine development.

Regionally, migraine burden showed significant heterogeneity across different SDI levels. Regional disparities in migraine are influenced by multiple factors related to healthcare access, lifestyle, and cultural perceptions (33, 34). In 2021, Low-middle SDI regions reported the highest absolute numbers for prevalence (31.19 million cases) and DALY (1.11 million cases). These figures likely reflect a combination of limited healthcare infrastructure, higher rates of underdiagnosis, and potential disparities in health-seeking behaviors. In comparison, high SDI regions demonstrated the highest incidence rates among females, which may be attributed to better healthcare access, more frequent diagnostic evaluations, and greater awareness of migraine as a clinical condition. However, it is important to note that the observed associations between SDI and migraine burden represent correlations, not causation. These patterns may be shaped by differences in surveillance systems, diagnostic practices, and sociocultural norms, rather than a direct effect of socioeconomic development. Enhanced awareness, improved diagnostic accuracy, and equitable access to effective treatments are critical, particularly in low- and middle-income regions (35, 36). Policymakers must also prioritize integrating migraine management into existing child health programs to mitigate the long-term health and socioeconomic consequences.

Notably, Western Sub-Saharan Africa experienced the most pronounced increases in all burden metrics over the past three decades, with EAPCs of 3.14 for prevalence and incidence and 3.17 for DALY. This trend underscores a rising health burden that may be exacerbated by under-resourced health systems, rapid population growth, and inadequate access to preventive and therapeutic care. Conversely, Central Europe recorded the steepest declines in prevalence, incidence, and DALY, likely reflecting the impact of effective public health interventions, improvements in healthcare infrastructure, and the widespread implementation of migraine management strategies.

While the GBD estimates reveal significant disparities in migraine burden at the national level, the interpretation of these differences must consider the influence of healthcare access, socio-economic factors, and national health policies (37), as well as the methodological nuances of the GBD study, including variations in data sources and imputation methods across countries. The GBD estimates indicate Brazil had a notably high ASIR of 4,111.61 per 100,000 and DALY rates (590.32 per 100,000) in 2021, suggesting a significant estimated health burden. However, interpreting the precise magnitude of these rates and comparing them directly with other countries requires consideration of the underlying data availability and modeling necessary for GBD estimates in different regions. Firstly, Brazil, despite being an emerging economy, faces challenges in terms of universal healthcare coverage and timely access to specialized care, particularly in rural and underserved regions (34). Additionally, the country’s diverse population with varying levels of healthcare access contributes to the underdiagnosis and undertreatment of chronic conditions like migraine (34). Thus, the high rates highlight the urgent need for improved migraine recognition and treatment at both the primary and secondary healthcare levels. School-based health education and screening programs have been effective in raising awareness and facilitating early diagnosis (38). Telemedicine platforms also offer scalable options for underserved regions with limited specialist access (39). Although Norway has a robust healthcare system, the increase could be linked to better identification and reporting of migraine cases in recent years, potentially reflecting greater attention to chronic conditions in an aging population (3). On the other hand, the decrease in DALY rates observed in Thailand (EAPC: −0.27) may be a result of successful public health initiatives, including advancements in treatment options and public health strategies. This observed trend based on GBD estimates should be interpreted with caution due to potential variations in data sources and collection methods over time in Thailand, and the precise contributing factors are likely multifactorial and cannot be definitively determined from the GBD dataset alone.

Dutch data showed lockdowns reduced monthly migraine days while improving well-being, suggesting positive behavioral adaptations (40). Italian studies noted mild migraine improvement during quarantines, potentially from resilient stress responses (41). Telemedicine implementation proved critical, with Hawaiian surveys demonstrating high patient satisfaction through ensured care continuity (42).

Females have a higher prevalence of migraine than males, likely due to a combination of biological, hormonal, and sociocultural factors (e.g., help-seeking behavior) (43–45). Biologically, the hormonal fluctuations associated with puberty, menstruation, and pregnancy are known to play a significant role in the onset and exacerbation of migraine in females (43, 44). During the 10–14 age group, when hormonal changes associated with puberty are most pronounced, migraine prevalence and incidence peak (45). Studies suggest that estrogen, which fluctuates during the menstrual cycle, plays a key role in this gender disparity, as female migraine sufferers often report attacks in relation to their menstrual periods (43). Adolescence is a critical period in which migraine can significantly affect school performance, social interactions, and overall quality of life (6). Additionally, sociocultural factors such as greater help-seeking behavior and reporting of symptoms may partly explain the higher diagnosis rates among females (6, 45). Recent large-scale cohort studies have shown that migraine typically begins before the age of 18, rather than in adulthood (46). This early onset highlights the importance of addressing pediatric and adolescent migraine burden in public health strategies.

In conclusion, our findings underscore the increasing global burden of migraine cases among children, which is characterized by significant regional, national, and demographic disparities. Addressing this challenge requires a multifaceted approach, including improved data collection, health resource allocation, and evidence-based interventions tailored to regional needs. Future research should focus on longitudinal studies to identify modifiable risk factors during critical developmental periods like puberty; developing and testing school-based prevention programs that combine nutritional interventions with stress-reduction techniques; and identifying biomarkers to differentiate migraine subtypes for personalized therapies. Additionally, establishing an international pediatric migraine registry could address data gaps in low-income populations, enabling tailored interventions informed by SDI-stratified analyses.

While previous studies have utilized GBD 2021 data to explore the global and regional burden of headache disorders among children and adolescents, our study offers a distinct and complementary perspective. Lu et al. (47) assessed the overall burden of headache disorders—including both migraine and tension-type headaches—but did not provide migraine-specific projections or stratifications below age 10. Moreover, the recent article by Steiner et al. (48) focused primarily on high-level advocacy, summarizing GBD findings with an emphasis on policy implications, but without conducting new epidemiological analyses or long-term projections.

In contrast, our study specifically targets the 5–14-year age group, a developmental window often underrepresented in migraine literature, and delivers burden estimates disaggregated by age, sex, SDI region, and country, alongside projections through 2050. This targeted approach provides a deeper understanding of pediatric migraine epidemiology, addressing an urgent gap in the literature and enabling tailored public health responses. To our knowledge, this is the first study to offer such detailed age-specific global estimates and forward-looking projections for pediatric migraine. These projections are based on the assumption of continued historical trends and do not account for unexpected global disruptions (e.g., pandemics, armed conflicts, climate change), which may alter future migraine burden trajectories.

### Limitations

This study has several limitations that should be considered when interpreting the findings. First, the exclusive focus on migraine (categorized as a level four condition in the GBD hierarchy) rather than broader headache disorders (GBD level three) creates inherent diagnostic specificity constraints. Second, substantial regional disparities in healthcare infrastructure introduce systematic surveillance biases, particularly affecting data reliability from low-resource settings. This highlights the heterogeneity and varying diagnostic accuracy in the GBD input data. In sub-Saharan Africa and South Asia, where pediatric neurology specialists are exceptionally scarce (averaging <0.3 per 100,000 children), diagnostic misclassification rates exceed 50% in primary care settings, coupled with severe neuroimaging access limitations (<18% availability in rural clinics) (49, 50). These structural deficiencies likely result in substantial under ascertainment of true migraine prevalence, particularly in populations experiencing healthcare transitions. Finally, the GBD framework’s methodological constraints require careful consideration, as case identification relies on heterogeneous data sources spanning hospital records, insurance claims, and national surveys that variably apply ICHD criteria, including the recent ICHD-3. Regional and temporal variations in adopting these diagnostic standards may affect case ascertainment and comparability of migraine estimates. This integration challenge is further complicated by temporal inconsistencies in data collection methodologies across the 31-year study period, potentially inflating prevalence trends in regions undergoing rapid diagnostic standardization. Our use of the BAPC model aligns with previous GBD forecasting studies and provides interpretable long-term projections. However, its assumption of linear trends may not fully reflect real-world non-linear factors (e.g., changes in healthcare access, global disruptions). Future research could consider alternative models (e.g., spline regression, machine learning) to enhance projection robustness. Furthermore, the variability in underlying data sources, diagnostic practices, and the necessity of data imputation in certain regions/countries inherent in the GBD methodology can affect the precision and comparability of estimates, particularly when making direct comparisons of absolute numbers, rates, or trends at the country level.

## Conclusion

This study underscores the growing global burden of migraine cases among children, with marked increases in its prevalence and significant disparities across SDI regions and countries. The High SDI regions demonstrated the highest female incidence rates, while the low-middle SDI regions bore the highest absolute prevalence burden. Notably, Western Sub-Saharan Africa experienced the steepest increases in migraine-related health metrics, emphasizing the need for targeted, evidence-based interventions. Future efforts should focus on addressing these disparities through improved resource allocation, comprehensive data collection, and region-specific healthcare strategies to mitigate the increasing impact of migraine in this vulnerable population.

## Data Availability

Publicly available datasets were analyzed in this study. This data can be found here: data used in this study can be found on GBD: https://www.healthdata.org/gbd.
