# Supplementary material for: Global trends and future projections of migraine burden in children aged 5 to 14 from 1990 to 2050
Source: Front Neurol. 2025 Sep 5;16:1641599. doi: 10.3389/fneur.2025.1641599 (PMC12446009; doi:10.3389/fneur.2025.1641599)
Supplement: Supplementary file 1 [file Table_1.docx]

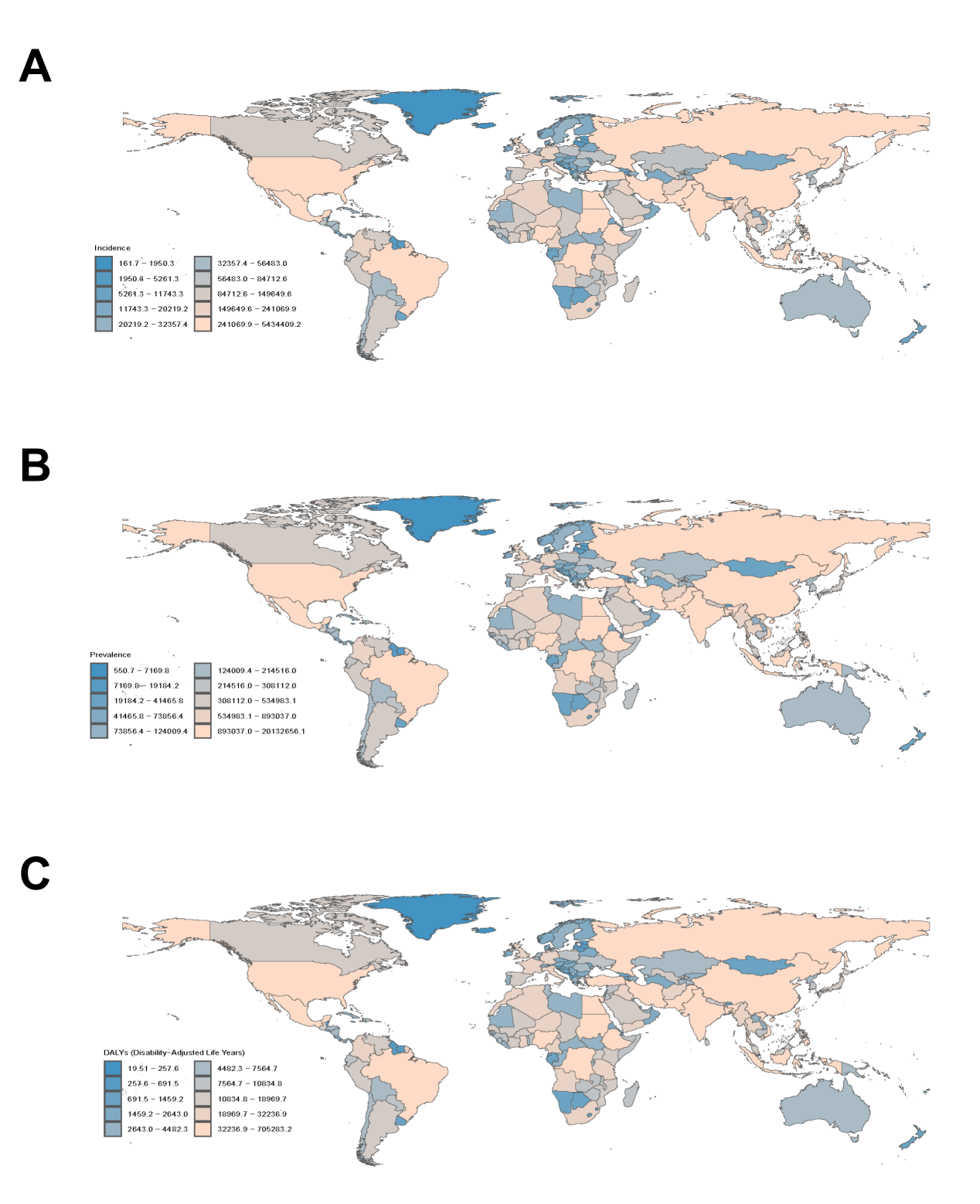


**Fig. S1** **A** The global disease burden of migraine incidence cases for both sexes in 204 countries and territories. **B** The global disease burden of migraine prevalence cases for both sexes in 204 countries and territories. **C** The global disease burden of migraine DALYs cases for both sexes in 204 countries and territories. Abbreviation: DALYs, Disability-adjusted life years.


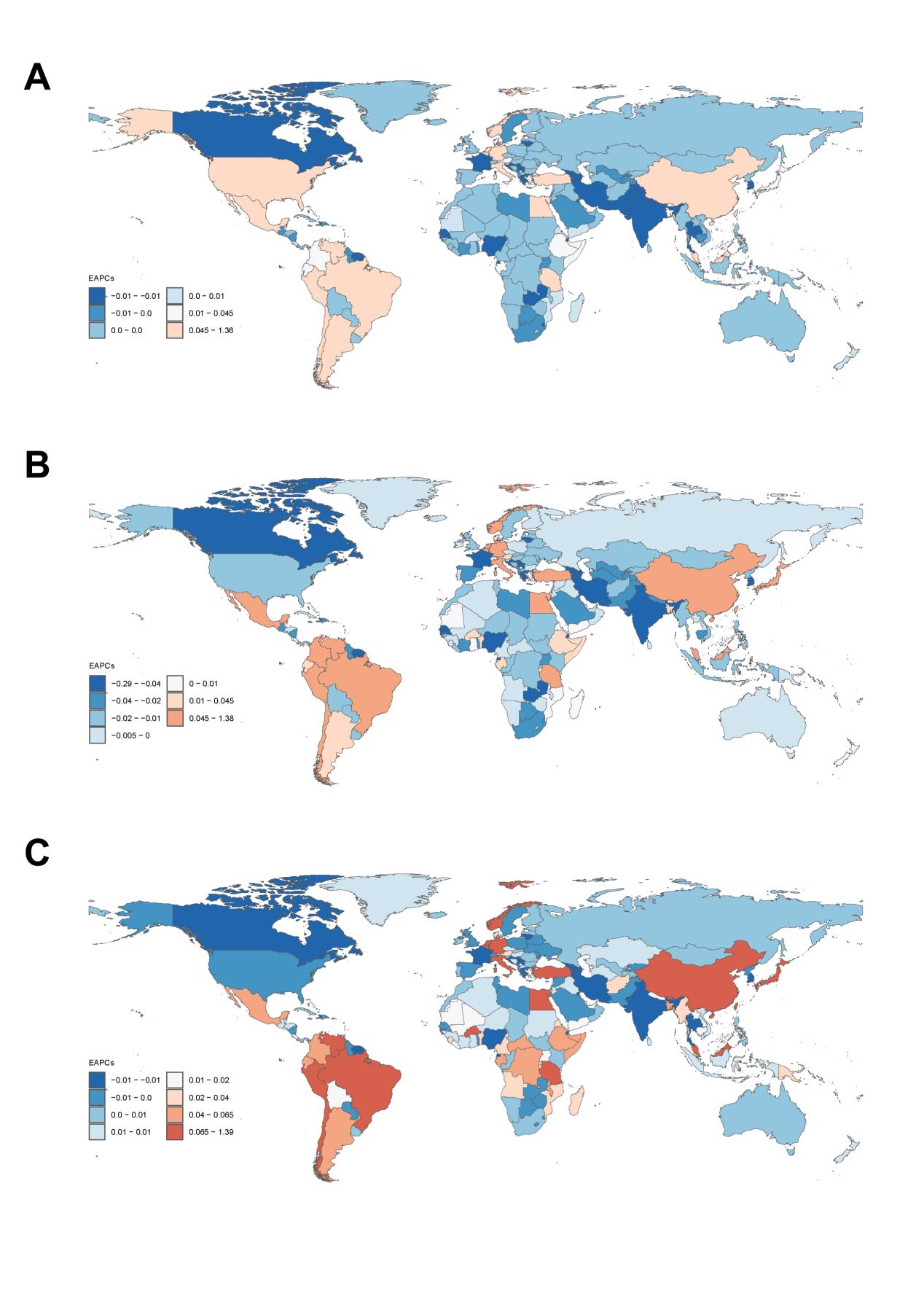


**Fig. S2** **A** The EAPC of migraine ASIR for both sexes in 204 countries and territories. **B** The EAPC of migraine ASPR for both sexes in 204 countries and territories. **C** The EAPC of migraine ASDR for both sexes in 204 countries and territories. Abbreviation: EAPC, Estimated Annual Percentage Change; ASIR, Age-standardized incidence rate; ASPR, Age-standardized prevalence rate; ASDR, Age-standardized disability-adjusted life years rate.
